# Mutations of the nACh Receptor M4 Helix Reveal Different Phenotypes in Different Expression Systems: Could Lipids be Responsible?

**DOI:** 10.3389/fphys.2022.850782

**Published:** 2022-05-04

**Authors:** Susanne M. Mesoy, Matthew Bridgland-Taylor, Sarah C. R. Lummis

**Affiliations:** ^1^ Department of Biochemistry, University of Cambridge, University of Cambridge, Cambridge, United Kingdom; ^2^ Clinical Pharmacology and Safety Sciences, Biopharmaceuticals R&D, AstraZeneca, Cambridge, United Kingdom

**Keywords:** Cys-loop receptor, M4, mutagenesis, aromatic interaction, nACh receptor

## Abstract

The role of the outermost helix (M4) in the pentameric ligand-gated ion channel (pLGIC) family is currently not fully understood. It is known that M4 is important for receptor assembly, possibly via interactions with neighboring M1 and M3 helices. M4 can also transmit information on the lipid content of the membrane to the gating mechanism, and it may form a link to the extracellular domain via the Cys-loop. Our previous study examining the α4β2 nACh receptor M4 helix using HEK cells indicated M4 here is more sensitive to change than those of other pLGIC. Many of these other studies, however, were performed in *Xenopus* oocytes. Here we examine the nine previously identified nonfunctional α4β2 nACh receptor M4 mutant receptors using this system. The data reveal that seven of these mutant receptors do function when expressed in oocytes, with only 2, the conserved Asp at the intracellular end of M4 and a Phe in the center, having a similar phenotype (nonfunctional) in both HEK cells and oocytes. The oocyte data are more consistent with studies in other pLGIC and demonstrate the importance of the expression system used. Of the many differences between these two expression systems, we suggest that the different lipid content of the plasma membrane is a possible candidate for explaining these discrepancies.

## Introduction

The nicotinic acetylcholine (nACh) receptor is the archetypal pentameric ligand-gated ion channel (pLGIC), a family of proteins responsible for fast synaptic transmission in the central nervous systems of both vertebrates and invertebrates (Cecchini and Changeux, 2015; Giastas et al., 2018). In the last decade, high-resolution structures of vertebrate, invertebrate, and bacterial pLGIC have shed light on the molecular details of these proteins and have begun to clarify their mechanism of action (Thompson et al., 2010; Nys et al., 2013; Nemecz et al., 2016; Plested, 2016), although it has been clear for many years that the receptors can be either homomeric or (more usually) heteromeric, with each of the five subunits possessing an N-terminal ligand-binding extracellular domain (ECD) and a transmembrane domain (TMD) with four membrane-spanning α-helices, M1-M4; eukaryotic receptors also have an intracellular domain. In the TMD, M2 lines the pore, M1 and M3 are adjacent to M2, and M4 and is the outermost lipid-facing α-helix.

**FIGURE 1:**
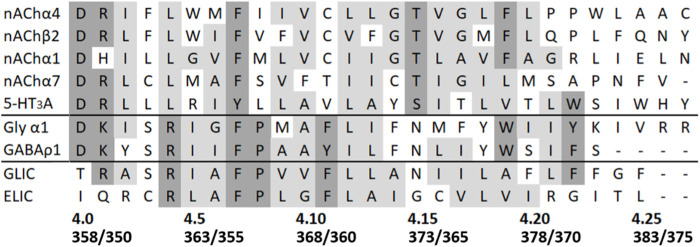
Sequence alignment of some example pLGIC M4 helices. Uniprot numbers are, in order, P09483, P12390, P02708, Q05941, Q8K1F4, P07727, P50572, Q7NDN8, and P0C7B7. Where residue types are predominantly conserved or identical they are coloured grey or dark grey, respectively. Residue numbers for the α4 and β2 nAChR subunits are shown below (α4/β2), along with the positional numbering system used here, where the conserved Asp at the intracellular end of M4 defines position 4.0.

There has been much interest in M4 recently, as despite the fact that it is physically distant from both the channel pore and the ligand-binding site, and it is not highly conserved (Figure 1), it has been shown to play a variety of roles including contributing to receptor activation e.g., (Hénault et al., 2015; Cory-Wright et al., 2018; Tang and Lummis, 2018; Mesoy et al., 2019). In addition, a variety of modulatory compounds such as ivermectin bind to or close to M4 e.g., (Althoff et al., 2014), supporting a functional role of this region of the protein.

An extensive investigation of the bacterial pLGICs, ELIC (*Erwinia* ligand-gated ion channel), and GLIC (*Gloeobacter* ligand-gated ion channel) showed that many Ala M4 substitutions are detrimental to channel function in GLIC, but beneficial in ELIC (Hénault and Baenziger, 2017). Similar experiments in anion-selective pLGIC M4s follow the GLIC pattern (Cory-Wright et al., 2018; Tang and Lummis, 2018), while the α7 nACh receptor broadly shows an ELIC-like pattern (da Costa Couto et al., 2020), as does the muscle nACh receptor α subunit (Thompson et al., 2020), although the latter contributes only two subunits to the heteropentamer. This led to the proposal that there are two different pLGIC archetypes, depending on the importance of TMD interactions versus M4 flexibility (Therien and Baenziger, 2017). The role of M4, however, may be more complex, as studies in cation-selective pLGICs expressed in *Xenopus* oocytes have shown M4 Ala mutations mostly cause a slight gain of function (e.g in α7 and muscle nACh receptors), yet reduce or abolish function when the receptors are expressed in HEK cells (e.g., in 5-HT_3_ and α4β2 nACh receptors).

Here we explore the apparent discrepancies between expression systems in α4β2 nACh receptors. The α4β2 nACh receptor is one of the major nACh receptor subtypes in the human brain, and here we are using it as a typical nACh receptor. Previously, we described how nine out of 28 double mutations in the α4β2 nACh receptor M4 abolished receptor function when expressed in HEK cells (Mesoy and Lummis, 2021). In this study, we find that the expression of seven of these nine mutant receptors in oocytes allows receptor function, and we suggest that the different lipids in these different expression systems could be responsible.

## Materials and Methods

*Molecular biology*: Site-directed mutagenesis using rat α4 (P09483) and β2 (P12390) nACh receptor subunits with an L9′A mutation (Fonck et al., 2005) was performed using the QuikChange site-directed mutagenesis kit (Agilent Technologies) and verified by nucleotide sequencing. Sequence identity between rat and human genes is 94% for β2 and 84% for α4, with the majority of differences between the α4 genes in the unstructured intracellular loop between helices M3 and M4, and no sequence differences within any of the transmembrane helices.

Genes were in pcDNA3.1 for expression in HEK cells, and in pGEMHE for expression in *Xenopus* oocytes; mRNA from the latter was produced using the T7 mMESSAGE mMACHINE kit (ThermoFisher).

*Cell culture*: Human embryonic kidney (HEK) 293 cells were maintained on 90 mm tissue culture plates at 37°C and 7% CO_2_ in a humidified atmosphere. They were cultured in Dulbecco’s Modified Eagle’s Medium/Nutrient Mix F12 (1:1) (Invitrogen, Paisley, UK) with GlutaMAX™ and 10% foetal calf serum.

*Expression in Xenopus laevis oocytes*: This was as described previously (Xiu et al., 2009; Thompson et al., 2011). Briefly harvested stage V-VI *Xenopus* oocytes were injected with 5–25 ng mRNA in the ratio 1:2 α4:β2 to obtain the high sensitivity (α4)_2_(β2)_3_ subtype (Tapper et al., 2004; Fonck et al., 2005). Electrophysiological measurements were performed 24–72 h postinjection in ND96 (96 mM NaCl, 2mM KCl, 1mM MgCl_2_, 5 mM HEPES, and pH 7.5). Nonresponsive receptors were assayed on at least two different days from multiple donors with or without chaperones, and oocytes from each donor were also tested with responsive receptors to ensure all sets of oocytes were capable of expressing functional channels.

*Electrophysiological recordings*: Two-electrode voltage clamp of *Xenopus* oocytes was performed using standard electrophysiological procedures with either a GeneClamp 500 amplifier or a Robocyte (Multichannel systems, Reulingeen, Germany). Glass microelectrodes were backfilled with 3 M KCl and had a resistance of approximately 1 MΩ. All experiments were performed at 0°C and a holding potential of –60 mV. Solutions of nicotine (Sigma) were prepared in ND96 and delivered to cells via a computer-controlled perfusion system.

*FlexStation™ recordings*: These were as previously described (Price and Lummis, 2005). Briefly, DNA was transfected into HEK293 cells at an α4:β2 ratio of 1:2 to obtain the high sensitivity (α4)_2_ (β2)_3_ subtype (Tapper et al., 2004; Fonck et al., 2005), and these were then incubated for 1–3 days before use. Blue fluorescent membrane potential dye (Molecular Devices Ltd., Wokingham, UK) was diluted in Flex buffer (10 mM HEPES, 115 mM NaCl, 1 mM KCl, 1 mM CaCl_2_, 1 mM MgCl_2_, 10 mM glucose, and pH 7.4) and added to cells on a 96-well plate. The cells were incubated at 37°C for 45 min and then fluorescence was measured in a FlexStation™ (Molecular Devices Ltd.) every 2 s for 200 s. Buffer or nicotine (1 nM - 1 μM) was added to each well after 20 s. NB EC_50_ values obtained from concentration responses curves using this technique are routinely lower than those obtained using voltage clamp data due to the different phenomena being measured i.e. dye partitioning into the membrane and changing fluorescence upon membrane depolarization versus direct current measurement.

*Radioligand binding*: This was undertaken as described previously (Thompson and Lummis, 2013) with minor modifications. Briefly, cell membrane samples were incubated in 0.5 ml Tris-HCl (50 mM, pH 7.4) with 1 nM [^3^H]-epibatidine (62.2 Ci/mmol, PerkinElmer, Boston, United States) for 4 h at 4°C. Nonspecific binding was determined using 300 µM nicotine, and radioactivity was determined by scintillation counting.

*Data analysis*: Concentration-response and radioligand binding curves were analyzed using Prism software (GraphPad, PRISM, San Diego, CA). Statistical analysis was performed using the ANOVA in conjunction with a Dunnett’s multiple comparisons post-test, or an unpaired *t*-test as appropriate; *p* < 0.05 was taken as statistically significant.

## Results

### Functional Characterisation of WT Receptors in *Xenopus* Oocytes

**FIGURE 2:**
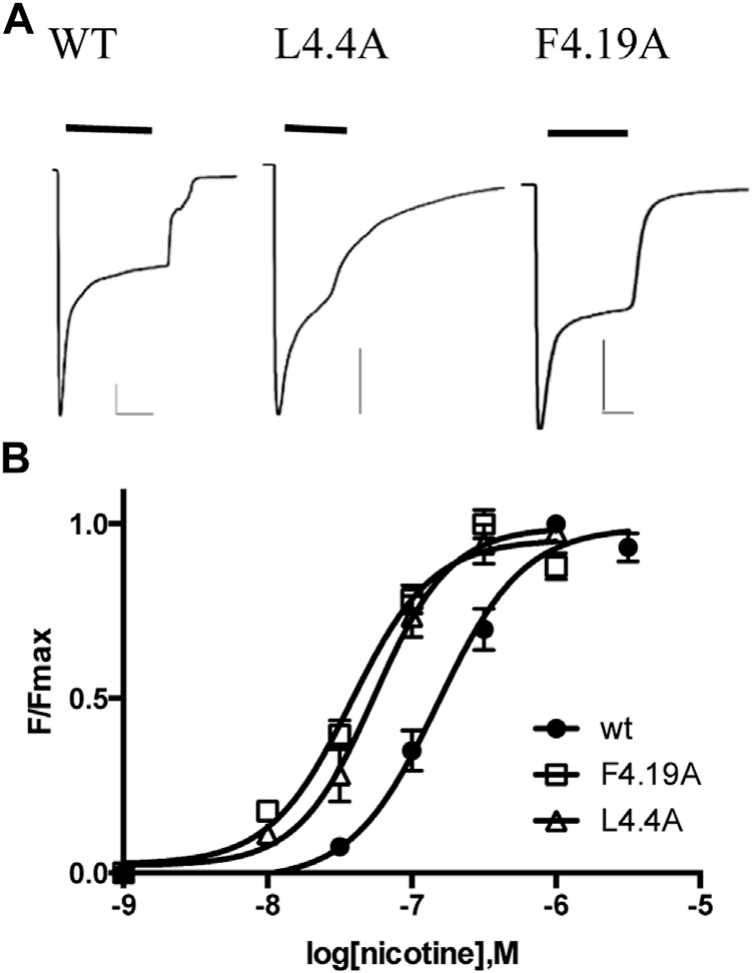
Characterization of α4β2 nACh receptors in *Xenopus* oocytes. **(A)** Typical currents elicited by 1 μM nicotine application to receptors expressed in oocytes. Scale bars are 500nA and 10s. **(B)** Concentration-response curves. Data = mean ± SEM, n = 3–4. Parameters derived from these data are shown in Table 1.

Our ‘wild-type’ α4β2 nACh receptors were those with an L9′A mutation, as we used previously in the HEK study (Mesoy and Lummis, 2021). This mutation has been previously shown to increase agonist potency but not alter pharmacology or kinetics (Mesoy and Lummis, 2021; Fonck et al., 2005; Tapper et al., 2004). Two electrode voltage clamp studies in oocytes showed concentration-dependent increases in current, with an EC_50_ of 165 nM (Figure 2; Table 1), a value similar to those obtained previously with the L9′A mutation (e.g., 80 nM (Xiu et al., 2009) and 230 nM (Fonck et al., 2005)).

**TABLE 1:** Functional parameters obtained from Ala substitutions of M4 residues.

Position (α/β)	Mutation	EC_50_ (nM)HEK^&^	pEC_50_ (M)oocyte	EC_50_ (nM)	n_H_	n
	WT	19	6.84 ± 0.05	165	1.4 ± 0.2	3
D358/D350	D4.0A	NF		NF		4
R359/R351	R4.1A	NF	7.06 ± 0.10	88	1.2 ± 0.3	6
F361/F353	F4.3A	NF	7.37 ± 0.08	42	1.7 ± 0.5	3
L362/L354	L4.4A	NF	7.30 ± 0.06	50	1.9 ± 0.4	4
F365/F357	F4.7A	NF		NF		4
L371/F363	L/F4.13A	6	7.04 ± 0.04	92	1.2 ± 0.1	4
G372/G364	G4.14A	101	6.96 ± 0.05	109	2.1 ± 0.5	4
T373/T365	T4.15A	NF	7.28 ± 0.10	52	1.2 ± 0.3	3
L376/M368	L/M4.18A	5	7.02 ± 0.07	95	1.5 ± 0.4	3
F377/F369	F4.19A	NF	7.43 ± 0.05	38	1.6 ± 0.3	4
P379/Q371	P/Q4.21A	NF	7.28 ± 0.06	54	1.4 ± 0.2	6
-/N376^$^	-/N4.26A	NF	7.11 ± 0.04	77	1.5 ± 0.2	8

Data are mean ± SEM. NF indicates nonfunctional receptors, where the addition of up to 1 μM nicotine had no effect. No parameters were significantly different from WT and ≥5-fold change, *p* < 0.05, and the 2-way ANOVA. ^&^data from Mesoy and Lummis (2021). ^$^The β subunit M4 is longer than that of the α subunit, so this residue in the β subunit has no α equivalent.

### Functional Characterisation of Mutant Receptors in *Xenopus* Oocytes

For these studies, we used receptors that had substitutions in both α and β subunits unless otherwise stated.

Our previous study of α4β2 nACh receptor M4 Ala mutants assayed in HEK cells revealed that nine were nonfunctional, despite eight of these being expressed at or above levels sufficient for detection of function (i.e., the expression level of the WT without chaperones, which shows robust responses in the functional assay) (Mesoy and Lummis, 2021). The data here reveal that 2 (D4.0A and F4.7A—see Figure 1 for numbering) of these nine mutant receptors were also nonfunctional in oocytes, but seven responded robustly to ligand application, giving similar trace shapes to the WT receptor (Figure 2; Table 1).

### Functional Characterisation of Receptors With Non-Ala M4 Mutations in HEK Cells

To further investigate the roles of the nine M4 residues crucial for α4β2 nACh receptor function in HEK cells, we assessed the effects of more conservative mutations at each of these positions. Some of these positions (e.g., 4.1) had strict residue requirements and tolerated few or none of the substitutions tested, while others (e.g., 4.26) tolerated a broad range of substitutions (Table 2).

**TABLE 2:** Functional parameters obtained following non-Ala substitutions of key M4 residues and expression of double mutant receptors in HEK cells.

Position (α/β)	Mutant	pEC_50_ (M)	EC_50_ (nM)	n_H_
	WT	7.74 ± 0.05	18	1.6 ± 0.2
	WT+	8.22 ± 0.04	6	1.1 ± 0.1
D358/D350	D4.0E+	NF		
	D4.0N+	NF		
	D4.0R+	NF		
	D4.0L+	NF		
R359/R351	R4.1K+	NF		
	R4.1E+	NF		
	R4.1S+	NF		
	R4.1Q+	NF		
	R4.1C+	NF		
	R4.1L+	NF		
	R4.1H+	NF		
F361/F353	F4.3L	7.49 ± 0.09	32	1.5 ± 0.4
	F4.3Y+	NF		
L362/L354	L4.4F+	7.96 ± 0.09	11	1.0 ± 0.2
	L4.4V+	NF		
F365/F357	F3.7Y+	7.85 ± 0.06	14	1.3 ± 0.2
	F3.7L+	NF		
T373/T365	T4.15D+	NF		
	T4.15S+	NF		
	T4.15C+	NF		
	T4.15V+	NF		
F377/F369	F4.19Y	7.38 ± 0.07	42	1.2 ± 0.2
	F4.19L	7.54 ± 0.09	29	1.3 ± 0.3
P379/Q371	P/Q4.21F+	NF		
WT/N376	WT/N4.26D	7.59 ± 0.08	26	1.8 ± 0.6
	WT/N4.26K+	7.30 ± 0.06	50	1.5 ± 0.3
	WT/N4.26C+	8.22 ± 0.4	6	0.9 ± 0.6
	WT/N4.26L	7.43 ± 0.1	37	3.6 ± 3
	WT/N4.26L+	7.59 ± 0.05	26	1.4 ± 0.2

Data are mean ± SEM. + indicates coexpression with chaperones, NF, indicates nonfunctional receptors. No parameters are significantly different from WT and ≥5-fold change, *p* < 0.05, and the 2-way ANOVA.

#### Interactions With M1/M3

**FIGURE 3:**
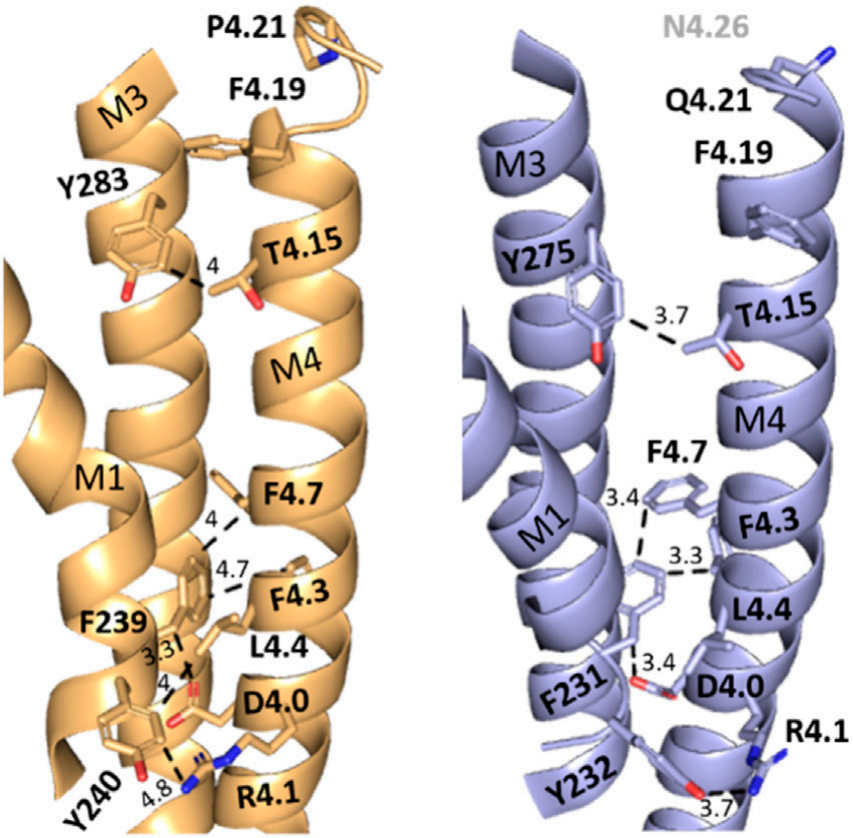
X-ray structure (5kxi) of human α4β2 nACh receptors in the closed state showing α4 (brown) and β2 (blue) transmembrane helices M1, M3, and M4. Selected M4 residues probed in this study and their possible aromatic interaction partners on M1 and M3 are shown as sticks. Distances along dashed lines in Å.

To probe if interaction with adjacent residues in M1 and/or M3 was important for function in HEK cells, we mutated residues on the M1/M3 interface that could act as interaction partners (Figure 3).

**FIGURE 4:**
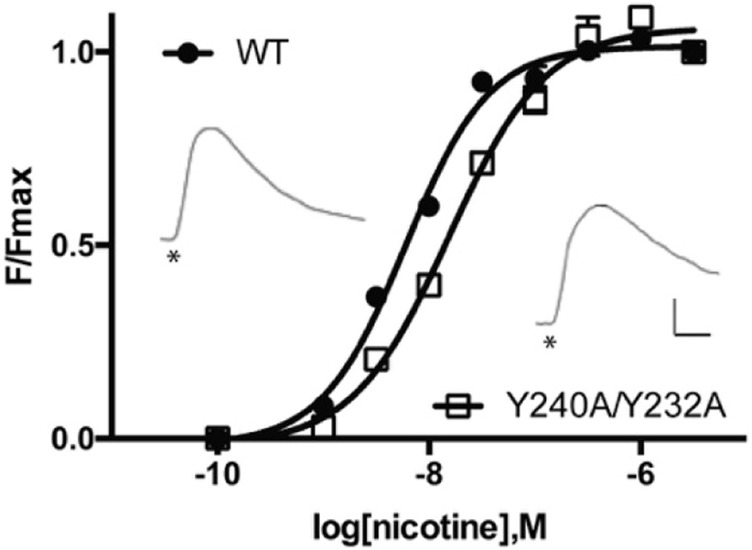
Concentration-response curves of α4β2 nACh receptors expressed in HEK cells. Data = mean ± SEM, n = 4. Functional parameters from these curves are shown in Table 2. Inset: typical responses to 300 nM nicotine applied at *. Scale bars = 40s and 50 arbitrary fluorescent units.

**FIGURE 5:**
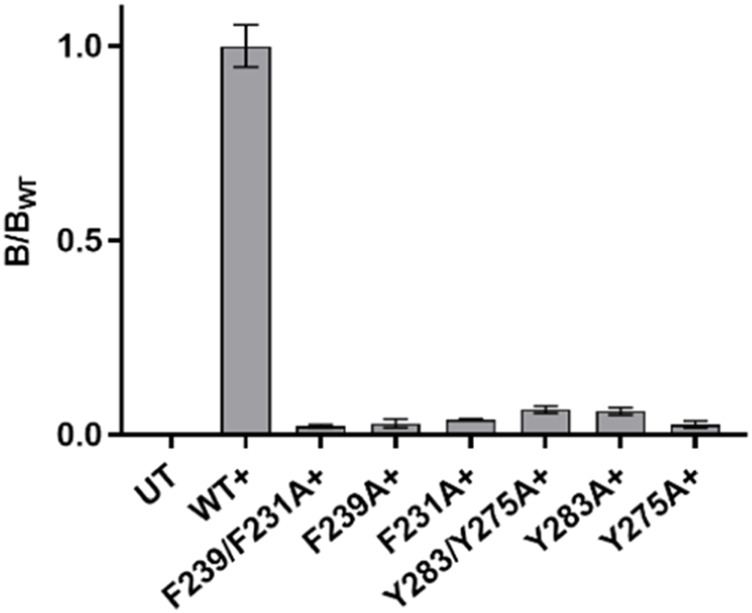
Specific binding of ^3^H-epibatidine relative to WT in single and double mutant nACh receptors in Table 3. Only the WT value is significantly different from untransfected (UT) cells. Data = mean ± SEM, n = 3–5. + indicates coexpression with chaperones.

There was no significant difference in functional parameters when Y240A/Y232A containing receptors were tested, but all other double mutants were nonfunctional, even when coexpressed with chaperones, and none were rescued by coexpression with a WT subunit (Figure 4; Table 3). Radioligand binding revealed none had levels of specific binding that were significantly different from untransfected cells, indicating they were not expressed (Figure 5). As this is a quite distinct phenotype to our M4 mutants, interactions here are unlikely to explain the HEK cell phenotype.

**TABLE 3:** Functional parameters obtained following Ala substitutions of M1/M3 aromatic residues and expression of mutant receptors in HEK cells.

Mutation (α/β)	pEC_50_ (M)	EC_50_ (nM)	n_H_
WT+	8.22 ± 0.04	6	1.1 ± 0.1
F239A/F231A+	NF		
F239A/WT+	NF		
WT/F231A+	NF		
Y240A/Y232A	7.84 ± 0.08	14	0.9 ± 0.1
Y283A/Y275A+	NF		
Y283A/WT+	NF		
WT/Y275A+	NF		

Data = mean ± SEM, n = 8. + indicates coexpression with chaperones, NF, nonfunctional receptors. Y240A/Y232A parameters are not significantly different to WT.

## Discussion

The aim of this study was to explore the effects of M4 mutations in α4β2 nACh receptors when the receptors were expressed in two different heterologous systems: *Xenopus* oocytes and HEK cells. In our previous study, 13 M4 mutants were initially found to be nonfunctional in HEK cells, a considerably higher number than observed for equivalent studies on other pLGIC M4s. Four of these could be rescued by co-expression with chaperones, suggesting that those mutations affect expression. Eight of the remaining nine could bind ligands, indicating a possible role in receptor function (Mesoy and Lummis, 2021). The data obtained here reveal that seven of these eight mutant receptors functioned when expressed in oocytes. Thus this study reveals that the expression system is fundamental to the observed effect of at least some pLGIC mutations, indicating that not only the amino acid composition but also the environment contributes to M4 behavior. These data explain the discrepancies previously observed between cationic pLGIC M4 helices, where some Ala mutations in 5-HT_3_A and α4β2 nACh receptor M4s can completely abolish channel function (assayed in HEK cells) (Mesoy et al., 2019; Mesoy and Lummis, 2021), whereas Ala mutations in other pLGIC are rarely inhibitory and often cause small gains in function (assayed in *Xenopus* oocytes) (Hénault et al., 2015; Cory-Wright et al., 2018; Tang and Lummis, 2018; da Costa Couto et al., 2020; Thompson et al., 2020) . Our data could also apparently explain discrepant studies showing that the C-terminal end of M4 can be deleted without affecting function in both ELIC (Hénault et al., 2015) and *Torpedo* nACh receptors (Tobimatsu et al., 1987) (data from oocyte expression), but other reports from receptors expressed in HEK cells suggest that cationic pLGICs do require the M4 C-terminus e.g., (Butler et al., 2009). We consider that the different lipid compositions may be responsible for these differences as discussed below.

### Lipids Modify pLGIC Behavior

Lipids have been known for more than 30 years to modulate pLGIC activity, and a range of studies on M4 has shown this helix acts as a lipid sensor, thereby influencing gating (Baenziger et al., 2000; Bouzat et al., 1998; Carswell et al., 2015; daCosta and Baenziger, 2009; Fong and McNamee, 1986; Nievas et al., 2008; Haeger et al., 2010; Lasalde et al., 1996; Lee et al., 1994; Rankin et al., 1997; Santiago et al., 2001). More recently it is becoming clear that certain lipids have particular importance for certain pLGICs, for example, purified nACh receptors tested in phosphatidylcholine + phosphatidylethanolamine bilayers will bind agonist but do not function (the so-called ‘uncoupled’ state), while phosphatidylcholine + phosphatidic acid + cholesterol will restore activity, and structures have revealed various bound lipids in GABA_A_ receptors, Gly receptors, GluCl and GLIC (Althoff et al., 2014; daCosta and Baenziger, 2009; Fong and McNamee, 1986; Criado et al., 1982; Hamouda et al., 2006; Hibbs and Gouaux, 2011; Huang et al., 2015; daCosta et al., 2013; Laverty et al., 2019). However to date, little importance has been given to the fact that *Xenopus* oocyte membranes have a higher cholesterol:phospholipid ratio (0.6–0.7 mol/mol) than HEK cell membranes (0.5 mol/mol), as well as more phosphatidylcholine (65% as compared to 35% of total phospholipids), but less sphingomyelin and a lower overall diversity of glycerophospholipids (precise numbers are not, as yet, generally agreed) (Opekarová and Tanner, 2003; Dawaliby et al., 2016). We suggest that cholesterol is a prime candidate to explain the differences we observe in the α4β2 nACh receptor, as cholesterol is important for nACh receptor function, and is known to bind to α4β2 nACh receptors: in the cryo-EM structure, PDBid 6CNJ 10 cholesteryl hemisuccinate moieties (modeled as cholesterol) are bound (Walsh et al., 2018). Insufficient interactions of cholesterol with our mutant receptors in a low cholesterol environment may mean that cholesterol is unable to bind, and thus the receptors could be retained in a nonfunctional, uncoupled state.

**FIGURE 6:**
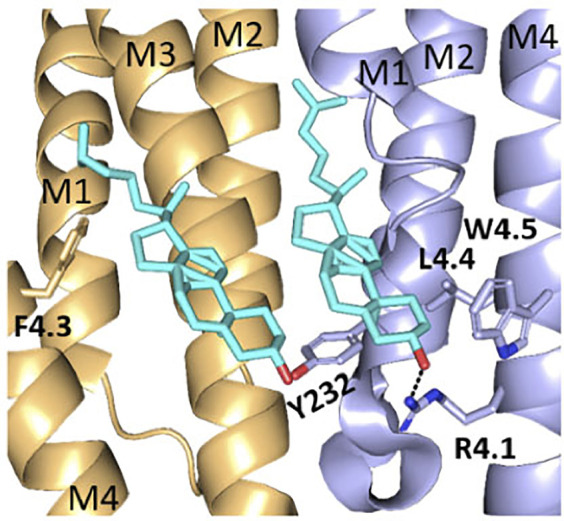
Cholesterol binding pockets. Two cholesterol moieties (cyan) binding to the α4 (brown) and β2 (blue) subunits in the nACh receptor (6CNJ). Shown in sticks are residues on M4 and M1 that may interact with cholesterol.

Several of the residues studied here are well placed to affect cholesterol interactions. For example: R4.1 is < 5Å from a cholesterol moiety in the α4β2 nACh receptor structure (Figure 6) with which it could H bond. A critical interaction here is supported by the fact that an R4.1A substitution abolished function when present in either β2 or α4 subunits. Furthermore evidence for a functional role for cholesterol comes from the 5-HT_3_ receptor: here, while an R4.1A mutation has no effect, a Y441A (4.7) substitution abolishes function but not expression (Mesoy et al., 2019), and an H-bond between cholesterol and Y441 on the M4 helix has been proposed as an essential part of channel gating (Guros et al., 2020).

Other residues, including F4.3 and βL4.4, contribute to hydrophobic regions adjacent to cholesterol and therefore could influence its binding. It is important, however, to avoid over-reliance on individual protein structures when considering potential lipid interactions with pLGICs, as these could vary between receptor states (closed, open, and desensitized). For example, recent molecular dynamics studies have shown that cholesterol can intercalate between M4 and M1/M3 of the glycine receptor when it is in the open state, but not in the closed state (Damgen and Biggin, 2020). Therefore residues that do not appear to be involved in lipid interactions in one structure cannot be excluded from consideration for a role in protein–lipid interactions or lipid modulation in another.

In addition to these residues at the intracellular end of M4, we have identified residues at the extracellular end which have a similar phenotype to those at 4.3 and 4.4 (nonfunctional in HEK cells, functional in oocytes). Possible reasons for the lack of responses of these mutant receptors in HEK cells are considered in our previous study (Mesoy and Lummis, 2021) where, for example, we suggest at T4.15 there is an H bond with an adjacent α-helix (T4.15 has been previously shown to affect gating kinetics in the muscle nACh receptor through a hydrogen bond (Bouzat et al., 2000)), and for 4.21 and 4.26 the structural data indicate specific interactions with residues in the ECD or TMD. We speculate that such interactions are critical in the absence of particular lipids, which could explain why these Ala mutants are nonfunctional in HEK cells. The current available structural data indicate these residues do not appear to contribute to cholesterol-binding, but data from other pLGICs are not inconsistent with their being involved or modulated by the binding of other lipids, e.g., a recent GABA_A_ receptor structure revealed phospholipid binding to the surface of the M1 and M4 helices on the extracellular side of the TMD (Laverty et al., 2019).

We also explored residues where Ala mutations increased (4.13 and 4.18) or decreased (4.14) the EC_50_ value in HEK cells; all of these had EC_50_ values similar to WT receptors in oocytes. If we assume that the differences here are also due to the different lipid environments of the receptors, then these data suggest that lipids do not only act as an on/off switch for these receptors but can also modulate their activity in either direction.

### Two M4 Mutants are Nonresponsive in *Xenopus* Oocytes and HEK Cells

D4.0A- and F4.7A-containing mutant receptors were nonfunctional when expressed in either HEK cells or oocytes. This is unsurprising for D4.0A—this Asp is well-known to be important for pLGIC expression, and Ala mutation disrupts this in other pLGICs in both HEK cells and oocytes (Mesoy et al., 2019; da Costa Couto et al., 2020; Lo et al., 2008). The role of F4.7 is less clear, although it is highly conserved in the M4 helix, being present in both cationic and anionic mammalian pLGICs as well as GLIC and ELIC. It is also the only residue in the 5-HT_3_ receptor M4 that is nonfunctional but expressed when mutated to Ala (Mesoy et al., 2019). There it was proposed that it forms a vital hydrophobic link between M4 and the adjacent α-helices and we suggest this may also be the case here.

### Other Factors

There are of course differences between HEK cells and oocytes other than the membrane composition, and these include protein expression levels, post-translational modifications, and intracellular factors. We have proven that the different phenotypes are not due to the elimination of expression in HEK cells, as eight of the mutant receptors studied here are expressed (Mesoy and Lummis, 2021), and intracellular factors or post-translational modifications have limited or no opportunity to alter M4 residues within the transmembrane domain. We, therefore, consider these differences unlikely to be an explanation for our data.

## Conclusion

In conclusion, we demonstrate here that of eight α4β2 nACh receptor M4 Ala mutants, which are expressed but nonfunctional in HEK cells, seven do function when expressed in *Xenopus* oocytes. A possible explanation for this is that these residues could either contribute to lipid interactions or that lipids could obviate the need for their interactions, and so the roles of these residues would differ in importance depending on the local lipid environment, which differs in HEK cells and oocytes. We suggest that being able to tune receptor activity by lipids may be useful at the synapse, possibly allowing large shifts in active channel populations simply through sequestration into different lipid environments.

## Data Availability

The original contributions presented in the study are included in the article/Supplementary Material, further inquiries can be directed to the corresponding author.

## Abbreviations

nACh, nicotinic acetylcholine; pLGIC, pentameric ligand-gated ion channel; ECD, extracellular domain; TMD, transmembrane domain; ELIC, *Erwinia* ligand-gated ion channel; GLIC, Gloeobacter ligand-gated ion channel; WT, wild-type; HEK, human embryonic kidney; *Uniprot protein IDs*, Rat α4 nACh receptor = P09483; rat β2 nACh receptor = P12390.
